# Novel DYRK1A Inhibitor Rescues Learning and Memory Deficits in a Mouse Model of Down Syndrome

**DOI:** 10.3390/ph14111170

**Published:** 2021-11-17

**Authors:** Wenche Stensen, Ulli Rothweiler, Richard Alan Engh, Melissa R. Stasko, Ilya Bederman, Alberto C. S. Costa, Anders Fugelli, John S. Mjøen Svendsen

**Affiliations:** 1 Department of Chemistry, UiT, The Arctic University of Norway, 9037 Tromsø, Norway; wenche.stensen@uit.no (W.S.); ulli.rothweiler@uit.no (U.R.); richard.engh@uit.no (R.A.E.); 2 Pharmasum Therapeutics AS, Gaustadalleen 21, 0349 Oslo, Norway; afugelli@pharmasum.com; 3 Departments of Pediatrics, Psychiatry, Macromolecular Science and Engineering, Case Western Reserve University, 11100 Euclid Avenue, Cleveland, OH 44106, USA; mxs1081@case.edu (M.R.S.); irb2@case.edu (I.B.); alberto.costa@case.edu (A.C.S.C.)

**Keywords:** protein kinase inhibitor, DYRK1A, down syndrome, neurodegeneration, learning and memory deficits, mouse models

## Abstract

Down syndrome (DS) is a complex genetic disorder associated with substantial physical, cognitive, and behavioral challenges. Due to better treatment options for the physical co-morbidities of DS, the life expectancy of individuals with DS is beginning to approach that of the general population. However, the cognitive deficits seen in individuals with DS still cannot be addressed pharmacologically. In young individuals with DS, the level of intellectual disability varies from mild to severe, but cognitive ability generally decreases with increasing age, and all individuals with DS have early onset Alzheimer’s disease (AD) pathology by the age of 40. The present study introduces a novel inhibitor for the protein kinase DYRK1A, a key controlling kinase whose encoding gene is located on chromosome 21. The novel inhibitor is well characterized for use in mouse models and thus represents a valuable tool compound for further DYRK1A research.

## 1. Introduction

Down syndrome (DS), first described by J. L. Down in 1866 [1], is a complex syndrome characterized by intellectual disability, distinctive facial features, low muscle tone (hypotonia), cardiac malformations, and leukemia in early infancy. In adulthood, DS is further complicated by additional health issues, such as early-onset Alzheimer’s disease [2]. Lejeune, Turpin, and Gautier established in 1959 the genetic basis for DS [3] as the presence of an extra copy of chromosome 21 (HSA21), which was the first time a genetic basis for an intellectual disability was identified. Although it is likely that the multifaceted phenotypic patterns of DS are the result of the action of several genes acting in complex molecular networks, this idea does not preclude that modification of specific targets in these pathways may deliver rescuing of some of the clinical manifestations of DS.

The *DYRK1A* gene is the human, high-sequence-similarity orthologue of the *Drosophila minibrain* gene [4]. Null mutations of the *minibrain* gene affects postembryonic neurogenesis leading to reduction of brain size. The *DYRK1A* gene is highly conserved in vertebrates [5] and encodes a dual-specificity tyrosine phosphorylation-regulated kinase, DYRK1A. This enzyme appears to play a role during brain development by regulating neurogenesis and neuronal differentiation in mice [6]. *DYRK1A* is highly expressed in the brain [6,7] where it plays an important role in the adult central nervous system manifested by the diverse learning and memory deficits observed in *DYRK1A* transgenic mice [8]. Individuals with DS are trisomic for the *DYRK1A* gene, and brain expression levels of DYRK1A are increased approximately 1.5-fold in areas such as the frontal, temporal, and occipital cortices, and the cerebellum, indicating that this protein is overexpressed in a gene dosage-dependent manner [9,10]. The DYRK1A protein kinase may hence be regarded as an archetypical dosage sensitive gene product, where its underexpression leads to Autosomal Dominant Mental Retardation 7 (MRD7) [11] and its overexpression contributes to the cognitive dysfunction in persons with DS. Mice overexpressing *DYRK1A* show severe impairment in spatial learning and memory indicating alterations in both hippocampal and prefrontal cortical function [12], effects similar to those found in murine models of DS. DYRK1A also induces tau phosphorylation inhibiting the biological activity of tau, primes tau-protein for further phosphorylation by glycogen synthetase-3β (GSK-3β) and promotes tau self-aggregation into neurofibrillary tangles (NFTs) [13]. The potential roles of DYRK1A on cognitive deficits in individuals with DS has recently been reviewed [14] suggesting that DYRK1A plays a key role in controlling cellular growth, neurogenesis and neuronal maturation. All the above observations implicate DYRK1A as a likely candidate for causing cognitive impairment in persons with DS.

In vivo models for DS are commonly murine based, although both *Drosophila* [4] and zebrafish [15] models are known. Mice have conserved synteny between chromosome 16 (MMU16) and the human chromosome 21 (HSA21) [16]. This shared synteny is the basis for mouse models of DS, such as the well-studied Ts65Dn [17] and the Ts1Cje [18] mice. The Ts65Dn has ≈104 genes that are orthologous with HSA21, while Ts1Cje has a shorter set of overlapping orthologous genes (≈84 genes), but both mouse models show learning deficits as assessed by different behavioral tests.

To further advance research on the role of DYRK1A in dementia and cognition in DS, there is a clear need to have access to tool compounds that are highly active and highly selective against DYRK1A, while simultaneously being compatible with the requirements of animal models. In this work we present the design and preparation of PST-001, a potent and very selective inhibitor of DYRK1A, that is non-toxic, orally available, with good pharmacokinetic properties including penetration of the blood–brain barrier enabling advanced in vivo studies in mice. In the present work we outline the design and development of PST-001 and verify its suitability in mouse models. Furthermore, by using the DYRK1A PST-001 inhibitor in Ts65Dn mice, learning and memory deficits in a contextual discrimination task seen in these animals can be rescued.

## 2. Results

**Figure 1:**
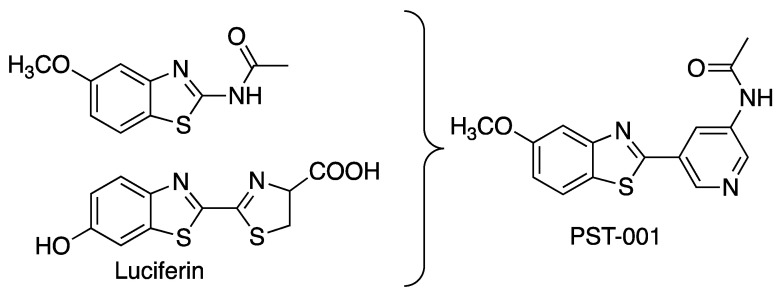
The design of PST-001.

The field of DYRK1A-active inhibitors has recently been extensively reviewed [19,20]. The first inhibitors described were harmine [21,22] and epigallocatechin gallate [23,24], but these have intrinsic problems, with the presence of PAINS motifs and potent MAO inhibition, respectively. Hence intense research for discovering new and more applicable inhibitors has been undertaken. The benzothiazole scaffold is among the more promising frameworks for DYRK1A inhibition, pioneered by the compounds INDY [25], luciferin [26], a library of fragment sized 2-acetamidobenzothiazole derivatives [27], and a series of thiazoloquinazolines [28,29,30]. The benzothiazole moiety appears to represent a privileged scaffold for DYRK-inhibition due to an unusual π-interaction between the benzothiazole sulfur atom in the inhibitor and the aromatic ring of the phenylalanine gatekeeper residue in the active site of the protein kinase [27]. A number of X-ray structures that accompanied the study of the 2-acetamidobenzothiazole fragments revealed multiple binding modes, but the common theme is that the substituent in the benzothiazole benzene ring interacts with Leu241 in the hinge region though hydrogen bonding, whereas the acetamido carbonyl group interacts with a salt bridge between Lys188 and Asp307. The latter interaction, however, is regarded as weak, as the molecules are too short to fully span the distance between the hinge and the salt bridge. Luciferin is another effective benzothiazole-containing inhibitor of DYRK1A, with a binding mode (determined on CDK2) resembling that of the 2-acetamidobenzothiazole fragments. In luciferin, the distance between the phenolic hydroxyl group and the carboxylate in the tricyclic molecule better meets the distance between the hinge and the salt bridge than for the benzothiazole fragments. With a phenolic hydroxyl group at the hinge and second arene group, its carboxylate moiety can interact more closely with the salt bridge. The dihydrothiazole ring in luciferin is regarded as insufficiently stable for use as an enzyme inhibitor, hence we replaced it with a chemically stable and similarly sized pyridine ring. A finding from the 2-acetamidobenzothiazole study was the position of the phenolic hydroxyl group in the benzothiazole, where the 5-position yielded more effective inhibitors than the 6-hydroxy analogs of luciferin. Using these structural clues, a molecule PST-001 was designed using the privileged benzothiazole scaffold (Figure 1), but with improved capacity to interact with the hinge and the salt bridge. The carboxylic acid moiety of luciferin was also replaced by an acetamido moiety, providing a molecule with calculated properties [31] compatible with oral availability (MW = 299.353, 5 H-bond acceptors, 1 H-bond donor, and clogP of 2.1246) [32].

**Figure 2:**
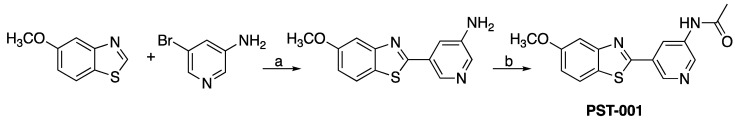
Preparation of PST-001. Reagents and conditions. a: Cs_2_CO_3_, Cu(I)Br, Pd(OAc)_2_, P(*t*-Bu)_3_, DMF, 150 °C, b: Ac_2_O, pyridine, CH_2_Cl_2_, rt.

PST-001 was prepared from 2-aminobenzothiazole through a direct palladium catalyzed coupling reaction with a bromopyridine forming 5-(5-methoxybenzo[d]thiazol-2-yl)pyridin-3-amine before the acetamido moiety was introduced as shown in the Figure 2 below. The preparation of PST-001 is described in detail in the Supplementary Information (experimental procedures and Figures S1–S6).

**Figure 3:**
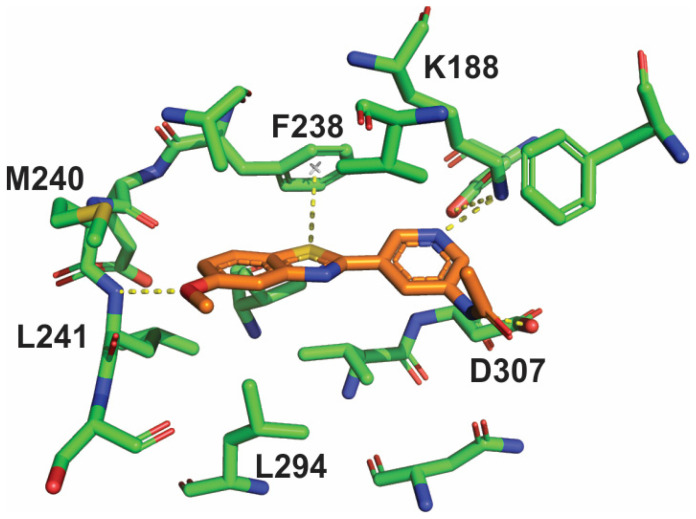
The binding mode, and main interactions of inhibitor PST-001 in the active site of the protein kinase DYRK1A as determined by X-ray crystallography.

An X-ray crystallographic investigation of the complex between PST-001 and DYRK1A (Supplementary Information, Table S1) revealed that the PST-001 molecule fulfilled its design goals (Figure 3). PST-001 is a type 1 protein kinase inhibitor, which binds to the ATP site of the active form of the enzyme. The methoxy oxygen atom in the inhibitor forms a hydrogen bond to the backbone NH of Leu241 in the hinge, whereas the acetamide NH on the pyridine ring forms a hydrogen bond to Asp307 and the pyridine nitrogen atom make a hydrogen bond to Lys188. The unusual π-interaction between the benzothiazole sulfur atom and the gatekeeper Phe238 is also evident as is an additional sulfur-π interaction between Met240 and the benzothiazole benzene ring.

The numerous binding interactions between PST-001 and DYRK1A translates to an effective inhibition of the DYRK1A enzyme with an IC_50_ value of 40 nM.

Selectivity within the protein kinase family was considered equally important to target binding for the purpose of developing a new DYRK molecular tool. PST-001 was designed on a DYRK privileged scaffold [27] and revealed an unusually high GINI-index [33] of 0.936 when tested at 1 μM (25 times the on-target IC_50_ value) on a 139-protein kinase panel (ATP concentrations were at or below the calculated K_m_ for ATP for each particular kinase). To put the selectivity of PST-001 in context, harmine has a GINI-index of 0.506, while pan-kinome inhibitor staurosporine has a GINI-index of 0.093 (with 10 uM ATP). Selected entries from the protein kinase profiling are shown in Table 1 (the full kinase profile tested at 1 and 100 μM is given in Supplementary Information Table S2), showing that the main off-targets were the other members of the DYRK-family and CLK2. (Aurora A seemed to be moderately activated.) Most important is the lack of inhibitory activity against GSK3β, a multifunctional protein kinase involved in phosphorylation of protein tau.

**Figure 4:**
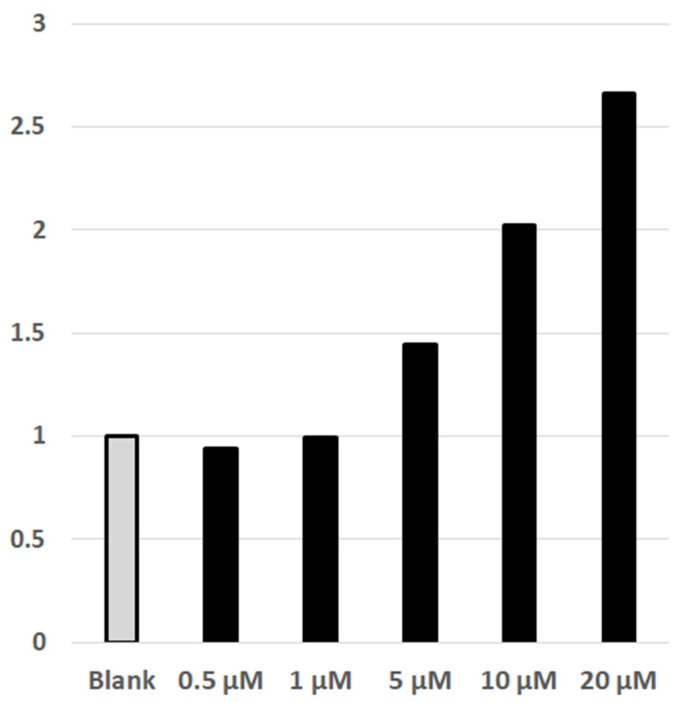
NFAT Luc reporter assay. The plot is normalized to the basal activity of the luciferase. Numbers indicate the fold of increase in luciferase activity upon inhibition of DYRK1A by PST-001.

The DYRK1A protein kinase is an intracellular and intranuclear enzyme, hence any effective inhibitor of DYRK1A must pass through both the cellular and the nuclear membranes. DYRK1A is involved in the NFAT/calcineurin signaling system [34], phosphorylating intranuclear NFATc. Upon phosphorylation, NFATc is trafficked out of the nucleus into the cytosol. The phosphorylated-NFATc in the cytosol may be dephosphorylated by Ca^2+^-activated calcineurin resulting in transport of the NFATc back into the nucleus. Inside the nucleus, NFATc forms a transcription complex with NFATn initiating the transcription of target genes. The involvement of nuclear DYRK1A in the NFAT/calcineurin system is utilized in a NFAT-Luciferin HEK293 cell assay. In this assay, the HEK293 cells are transfected with NFAT and DYRK1A plasmids such that the NFATc-phosphorylation via DYRK1A predominates over dephosphorylation of extranuclear phosphor-NFATc by calcineurin activated by Ca^2+^. In this situation, the NFAT-transcription complex will not be formed, and the luciferase gene will not be transcribed. If, however, DYRK1A is inhibited, reducing phosphorylation of NFATc, the NFAT-transcription complex will form, and the reporter luciferase will be produced [35]. The assay thus shows increasing luciferase activity provided the inhibitors are able to enter the cell nucleus and inhibit the elevated amounts of DYRK1A effectively. Figure 4 shows dose dependent luciferase activity as a function of PST-001 concentration, showing that PST-001 can enter the nucleus of the HEK293 cells and inhibit DYRK1A enzymatic activity when present at μM levels in the cell media. The exact IC50 value could not be determined as upper limit of luciferase activity could not be reached due to the limited solubility of PST-001 in the assay system.

The demonstration that PST-001 is able to modify the activity of intranuclear DYRK1A is a necessary but not sufficient demonstration of its utility as a tool compound in animal models. In order to assess results from in vivo experiments detailed knowledge of the ADME-properties of PST-001 is needed. The pharmacokinetic properties of intravenous administration of PST-001 was investigated in a rat model. A bolus injection of PST-001 at a dose of 1 mg/kg in the tail vein revealed a maximum venous concentration of 647 ng/mL after 2 min (Table 2). The compound rapidly disappeared from circulation with a t_½_ of 43 min. No PST-001 could be found in the urine, indicating that rapid metabolism is the main cause of the short t_½_ observed. A separate mouse microsome stability assay of PST-001 showed a half-life (t_½_) of 25 min, corresponding to an intrinsic clearance, C_int_, of 55 μL/min/mg, regarded as high. Bolus injection of PST-001 in the tail vein of a mouse confirmed a rapid clearance from blood plasma with a t_½_ of 0.15 h and C_max_ of 362 ng/mL obtained 5 min after the injection. In the mouse model, the PK-parameters were also determined in the brain samples. In the brain PK, t_½_ and T_max_ were identical to the values observed in plasma; however, C_max_ were 867 ng/mL, more than the double than in plasma. Oral administration of PST-001 in the mouse model at a fivefold dose compared to the IV experiments showed a maximum plasma concentration (C_max_) of 76 ng/mL after 1 h (T_max_) representing a bioavailability, f, of 21%. Samples of brain tissue were taken at the same time-points as the plasma samples and showed that the concentrations of PST-001 in the brain tissue was the double of that of the plasma concentration, hence indicating that PST-001 is transported efficiently across the blood brain barrier both by IV and PO administration (Table 2). Stable oral bioavailability of PST-001 was established through twice-a-day administration in the animal food of 10 mg/kg of PST-001, giving a steady state brain concentration of 150 ng/mL and a plasma level of 76 ng/mL (corresponding to 0.25 μM).

**Figure 5:**
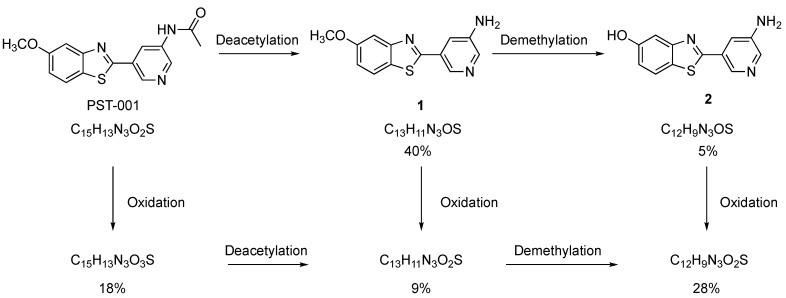
Identified metabolic pathways of PST-001. The percentages represent a snapshot of the distribution of the metabolites at a 30% conversion of PST-001.

The results from the pharmacokinetic study of PST-001 in rats suggests rapid metabolism may be the cause of the short t_½_ observed. Effective transport to the brain acting as a reservoir may also contribute to the rapid plasma clearance of PST-001. A short t_½_ is in itself not detrimental to animal studies, but a rapid metabolism creating metabolites with on- or off-target effects would handicap interpretation of the results. Hence, we included studies of which metabolites are formed at what amounts, along with their DYRK1A activities. First an in silico assessment of the site and type of cytochrome p450 (Cyp) metabolism was undertaken using the SMARTcyp [36] and GLORY [37,38] metabolite prediction services. SMARTcyp predicted that the three main sites for Cyp-metabolism were the C-5 methoxy methyl, the benzothiazole sulfur atom and the methyl group of the acetamido moiety. These predictions were further substantiated by the GLORY metabolism web-service, where the three most probable metabolites were predicted to be the demethylated 5-hydroxy-analog of PST-001, hydroxylation of the acetamide methyl group yielding the glycolic amide derivative of PST-001 and oxidation of the benzothiazole sulfur atom. To test the in silico predictions, PST-001 was subjected to a 1 h treatment with rat liver microsomal fraction (RLM) [39]. A high-resolution LCMS target analysis verified the presence of the suggested metabolites. The analysis was performed using an internal standard allowing for calculation of the mass balance in the metabolism study. The sum of all analytes (PST-001 and the suggested metabolites) was close to 100%, suggesting that the set of identified metabolites represents a quantitative picture of the metabolism of PST-001. As shown in Figure 5, the metabolism takes place through two different primary processes; Oxidation of PST-001 (S-oxidation or acetamido hydroxylation, which are indistinguishable by mass spectrometry) forming molecules with the formula C_15_H_13_N_3_O_3_S or hydrolysis of the acetamido group (deacetylation) forming compound **1**. The latter process is likely not a Cyp reaction, but rather a result of protease activity in the RLM fraction. The predicted demethylated metabolite of PST-001 predicted by SMARTcyp was not detected. The primary metabolites (oxidation metabolite C_15_H_13_N_3_O_3_S and compound **1**) were further metabolized to secondary metabolites by deacetylation or oxidation, respectively forming a secondary metabolite with formulas C_13_H_11_N3O_2_S. Compound **1** is also metabolized by a demethylation to compound **2**. The final metabolite(s) is a compound with formula C_12_H_9_N_3_O_2_S where all three processes had taken place. The investigated metabolite mixture had satisfactory mass balance indicating that the major metabolites and pathways have been identified.

It is also highly likely that several of the metabolites are active inhibitors of DYRK1A. Deacetylation to compound **1**, an intermediate in the preparation of PST-001, was profiled in the protein kinase panel and showed high selectivity towards DYRK1A and inhibitory activity similar to PST-001. The X-ray structure of PST-001 does not show any critical interaction with the acetyl group (Figure 3); and deacetylation would not significantly change the hydrogen bond donor capacity of the amino group. Similarly, potential acetamido hydroxylation would not eliminate essential interactions, and the additional hydroxyl group could be hydrogen bonded to water or polar protein groups. Compound **2** formed by demethylation of compound **1** may seem more likely to change binding properties, since this changes the details of critical hinge H-bond interactions with Leu241NH. However, our previous studies of fragments bound to DYRK1A included direct comparisons of 5-methoxy- and 5-hydroxybenzothiazole compounds. These indicate that the desmethyl metabolite of PST-001 would likely show similar activity against DYRK1A [27].

Considering the interactions of the inhibitor with the DYRK1A target (Figure 3), only the possible oxidation of the sulfur atom would be clearly disruptive to binding by disabling the sulfur-π interaction that is key to the selective and efficient binding to the target protein kinase. The total amount of oxidated metabolites is approximately 50% representing an upper limit of metabolites where the target affinity may be more compromised.

**Figure 6:**
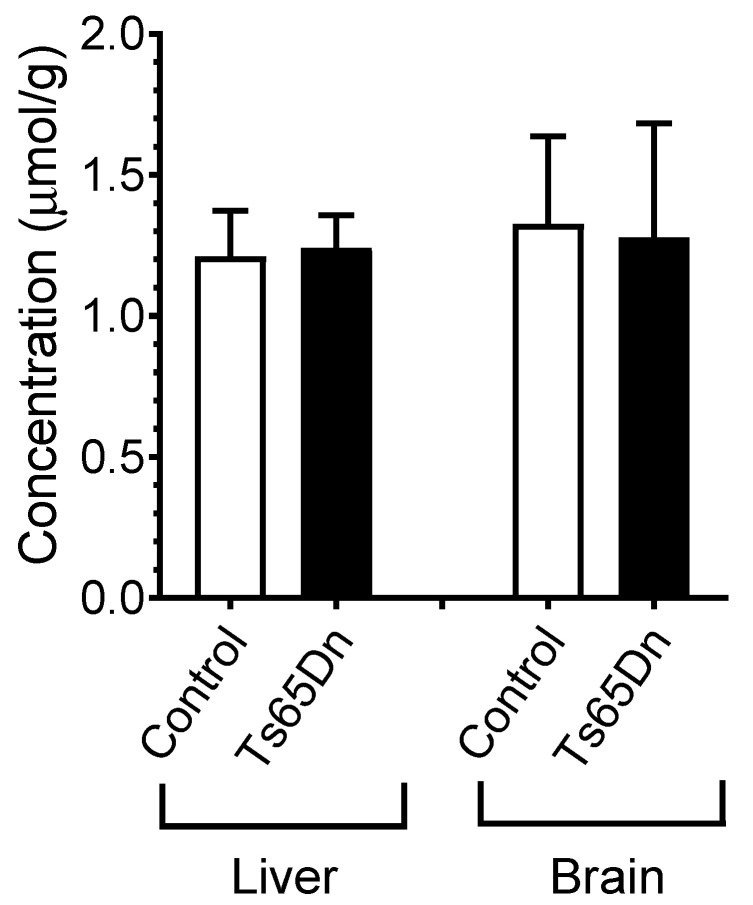
Levels of PST-001 found in the brain and in plasma after completion of the fear conditioning experiment.

Given that PST-001 is orally active, allowing stable plasma levels in mice to be obtained by normal feeding, a dose escalation study was performed in order to secure an effective dose of PST-001 in the brain of the Ts65Dn mice. The mice were fed with a mouse-palatable food mixture containing 35 mg, 50 mg, and 100 mg PST-001/kg powdered food and the steady state level in the liver and the brain was monitored by LC/MS. The highest dose provided a continuous concentration of 1.25 μmol/g both in the liver and in the brain and was used in fear conditioning experiments. There was no difference in the PST-001 concentration between control and Ts65Dn mice (Figure 6). This was confirmed by Two-way analysis of variance (ANOVA), which did not detect any significant genotype (F(1, 36) = 0.0061, *p* = 0.9380) or tissue (F(1, 36) = 0.3706, *p* = 0.5465) effects on the measured PST-001 concentrations.

The capacity for contextual memory of 10- to 16-week old Ts65Dn mice was compared with age-matched euploid control mice using a fear conditioning protocol [40]. Briefly, the mice were exposed for 3 min to the context test chamber serving as a conditioning stimulus, followed by an electric shock for 2 s representing the unconditioned stimulus. The memory test was performed 24 h later, by re-exposing the mice to the same context. Freezing, defined as a species-specific defensive reaction characterized by lack of movement besides respiration and heartbeat, associated with crouching posture, was used as a measure of learning, and assessed for 180 s, every 9 s by two trained observers aware of the mouse genotypes and the pharmacological agent being used.

**Figure 7:**
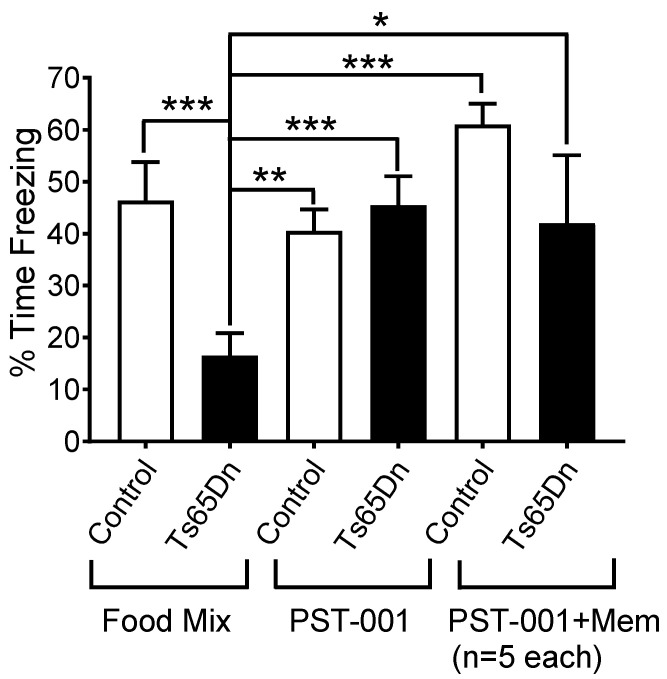
Contextual fear conditioning model. Parallel experiments showing the effect of PST-001 in the food on the freezing time in the contextual fear conditioning model and the combined effect of PST-001 in the food and acute memantine injections. Data represent mean ± SEM and statistical significance is expressed as *, ** and ***, for *p* < 0.05, *p* < 0.01, and *p* < 0.001, respectively.

As can be seen in Figure 7, PST-001-treated Ts65Dn mice displayed freezing at a comparable percentage to both placebo-treated (food mix without PST-001) and PST-001-treated control euploid animals. We also performed parallel preliminary tests of the combination of a chronic treatment of PST-001, as described above, and two acute I.P. injections of memantine hydrochloride (5 mg/kg of mouse weight) 15 min before shock exposure and 15 min before exposure to the context chamber 24 h later. The mechanism of action of PST-001 is expected to be different from the mechanism of action of the drug memantine, and one could potentially envision a scenario in which both PST-001 and memantine might be used together in the same individual. In these preliminary tests we observed that PST-001 treatment, even when in combination with memantine, eliminated the learning and memory deficits displayed by Ts65Dn mice as assessed by contextual fear conditioning.

Factorial ANOVA (performed with Statistica Academic version 13, TIBCO Sofware, Palo Alto, CA) revealed that both genotype (F(1, 46) = 6.8950, *p* = 0.0117) and drug treatment (F(2, 46) = 4.2587, *p* = 0.0201) had significant effects on the percentage of freezing during the context test. In addition, a significant interaction between genotype and drug treatment was detected (F(2, 46) = 4.5596, *p* = 0.0156), which indicate a genotype-dependent drug response. Post hoc (Fisher LSD) tests confirmed that, between the two groups that had the food mixture without any medication, euploid control mice displayed a significantly larger percentage of freezing during the context test than Ts65Dn mice (*p* = 0.0007). In contrast, Ts65Dn mice that received PST-001 treatment showed no significant difference in freezing behavior compared with untreated control (*p* = 0.9107) or PST-001-treated control mice (*p* = 0.5517). Therefore, PST-001 completely eliminated the learning and memory deficits displayed by Ts65Dn mice as assessed by contextual fear conditioning. Interestingly, this compound produced no detectable effects on the percentage of freezing behavior displayed by control euploid mice (*p* = 0.4815). Post hoc tests also showed that Ts65Dn mice that received combined PST-001 and memantine treatment showed no significant difference in freezing time compared with untreated control (*p* = 0.6708), PST-001-treated control mice (*p* = 0.8855), or control mice treated with combined PST-001 and memantine (*p* = 0.1190).

Preclinical experiments of the effects of compound PST-001 in the mouse model of DS Ts65Dn demonstrated that the compound is well tolerated by these mice and that chronic oral administration of PST-001 eliminates context discrimination behavioral deficits. We have confirmed the oral availability and brain penetration of this compound in mice. Preliminary experiments involving the co-administration of PST-001 and memantine failed to show additive or antagonistic interactions between these two pharmacological agents.

## 3. Discussion

The protein kinase DYRK1A has in the last decade moved from relative obscurity to become a widely studied potential target for drug development. DYRK1A regulates important cellular processes, in particular proliferation and differentiation of neuronal progenitor cells [41], as well as neurodegeneration [42]. It is thus no surprise that neurological syndromes and disorders such as DS [14,34,43,44,45,46,47,48,49,50,51,52,53,54], Alzheimer’s disease [44], Parkinson’s [55,56,57], and Pick’s disease [58] all involve abnormal expression of DYRK1A [59]. Abnormal DYRK1A expression is also linked to a raft of malignancies like glioblastoma multiforme [60], gastrointestinal stromal tumors, lung cancer [61], melanoma, and leukemias (AML, AMLK, and ALL) [62]. Furthermore, DYRK1A may represent a target for the treatment of diabetes Type 1 and 2, as the kinase is involved in pancreatic β-cell proliferation [63].

As a consequence of the increased interest in therapeutic modification of DYRK1A activity, a wide range of inhibitors has been reported. Few of these inhibitors have an established selectivity profile, which limits their utility as tool compounds. Furthermore, reliable molecules for animal studies must also have acceptable and known pharmacokinetic properties, preferably including good blood–brain passage, for reliable interpretation of results.

The first DYRK1A inhibitor tested in animal models was INDY, developed by Ogawa et al. [25] INDY showed effects in biochemical and cellular models and was also applied on *X. laevis* embryos with *xDYRK1A* overexpression-induced deformity, where the compound rescued the deformity in the eye and head of the embryos. The next inhibitor tested was epigallocatechin-3-gallate (EGCG). In 2009, it was reported that major features of partial trisomy of MMU16 (a model of HSA21 trisomy) found in transgenic phenotype mice overexpressing DYRK1A could be rescued by feeding these animals with polyphenol-based diets from gestation to adulthood [24]. In 2014, a green tea extract was administered to Ts65Dn mice for a month, and supposedly the EGCG contained in the extract normalized the DYRK1 activity in hippocampus, and significantly improved their performance in the Morris water maze test [23]. However, although the authors did find signals of improved cognition in a pilot clinical trial, the effect sizes of these improvements were small, and were not statistically significant without a concomitant cognitive training program being applied to the study participants [64]. The tool compounds GNF7156 and GNF4877 used in a study of induction of beta-cell proliferation are non-selective DYRK1A and GSK3β inhibitors making interpretation of the results difficult [65].

A recent review characterizes the main challenges of pharmacologically inhibiting DYRK1A and targeting trisomy as the bridging of two knowledge gaps: (1) the lack of understanding of the downstream targets of excessive DYRK1A activity leading to the typical phenotypic signs of trisomy 21; and (2) the bioavailability, specificity, and dose-dependent inhibition of DYRK1A by candidate inhibitors must be ascertained for specific tissues, and correlations between pharmacological actions and therapeutic outcomes need to be established [66]. With this background, we designed PST-001 as a tool compound for studying in vivo effects of DYRK1A inhibition. The important design criteria include high target activity, high specificity with few and well characterized off-target binding, ability to reach the DYRK1A target in the relevant tissue, including the brain and being able to administer perorally.

PST-001 is a molecule designed to be a selective DYRK-family inhibitor. The benzothiazole part originates from the finding that luciferin is an inhibitor for a select few protein kinases [26], in particular the DYRK-family, but also CK2, Aurora A and B as well as CDK2/Cyclin A. The effects of substitutions pattern on the benzothiazole ring were explored using a series of 2-acetamidobenzothiazole fragments and an explanation for the unusual high DYRK-selectivity was offered in a subsequent experiment [27]. The key to the uncommon selectivity of the fragment sized molecules was proposed to be an interaction between the benzothiazole sulfur atom and F238 through a sulfur–π interaction. The selectivity of these short fragments was surprisingly high, strongly favoring the intended target, but the on-target potency was inadequate and had to be increased for the compounds to be applicable tool compounds for in vivo studies. Examination of the structures from X-ray crystallographic studies pointed out that the fragments should be longer to span the active site of DYRK1A from the hinge to Lys188. Inspired by the binding mode of the analogous benzothiazole derivative, luciferin, which does span the active site via its additional dihydrothiazole ring, it was decided to lengthen the benzothiazole fragment by using a heteroaromatic structure, including the choice of a heteroatom to interact with Lys188. A *meta*-substituted pyridine ring was selected on the basis that the basic nitrogen atom in the pyridine ring would work as an acceptor to the lysine side chain ammonium ion. Furthermore, a *meta*-relationship between the benzothiazole and the pyridine nitrogen atom would place the nitrogen in a geometrically advantageous position for an interaction with the ubiquitous Lys-residue. Finally, an acetamido group was introduced in the other meta-position of the pyridine ring to interact with Asp307, and thereby securing the orientation of the pyridine nitrogen “inwards”, facing Lys188. The presence of two lipophilic residues, Leu294 and Val308 creates a lipophilic floor in the DYRK1A ATP-binding pocket fitting snugly to the lipophilic aromatic core of PST-001. PST-001 showed high potency against DYRK1A and retained the unusually high selectivity typical for the benzothiazole fragments. Importantly, the kinase profiling revealed that there is no discernible activity against GSK3β at concentrations where DYRK1A is fully inhibited, a common limitation known from typical DYRK1A inhibitors such as INDY [25] and the leucettamine derivative L41 [67].

DYRK1A is an intranuclear protein kinase and tends to accumulate in nuclear structures [68]. Transport across cellular and nuclear membranes is thus a critical requirement for effective inhibitors One of the intranuclear targets for DYRK1A phosphorylation is the nuclear transcription factor NFATc1 [69]. The established NFAT/calcineurin reporter system is thus an appropriate choice for monitoring the intranuclear activity of DYRK1A [34]. We adapted the HEK-cell reporter system and showed that PST-001 has the ability to pass the cellular envelope as well as entering the cell nucleus and inhibit intranuclear DYRK1A.

The next level of complexity for a successful in vivo model is to ensure that the inhibitor reaches the intended tissue where the effect is expected to take place. Regarding the pharmacological effects of DYRK1A inhibition, the intended tissue may vary from inside the brain as in the case of neurodegenerative diseases and CNS cancers such as GBM, via the pancreas for β-cell proliferation, to the general circulation for metastatic cancers in the lungs and intestines. It is hence important to establish the pharmacokinetics of the inhibitors in relevant animal models, as well as gaining insight regarding key ADME parameters in the animal model.

Upon administration of PST-001 to the animal (typically a rodent like a mouse or a rat), either through an IV or PO route, the pharmacokinetic measurements reveal that the molecule is rapidly distributed in plasma, with a T_max_ of 2 min and 15 min for IV and PO administration, respectively. Peroral administration is effective and approximately 25% of the IV-plasma C_max_ can be obtained through a PO administration of the same dose. The lifetime of the molecule in plasma is short, however, with t_1/2_ in the range of less than 1 h. On the other hand, brain penetrance is good, with brain levels reaching 50% of the plasma level at T_max_ after PO administration.

As indicated by the short t_1/2_, PST-001 is rapidly metabolized to defined metabolites, all of which may be active inhibitors against the DYRK1A target.

As a proof of concept, an in vivo study in Ts65Dn mice was undertaken. The end point of this study was improvement in learning and memory, as measured by the animals’ performance in a context discrimination task. A continuous feeding of 100 mg PST-001/kg in the animal food produced a steady state level of 1.25 μmol/g PST-001 in both plasma and brain tissue in control mice as well as Ts65Dn mice. Before the contextual fear conditioning experiments, all mice were exposed for three-days to either food mixture alone or food mixture with added PST-001, and exposure continued during the experiment.

The contextual fear experiments showed that there is a large and statistical difference in learning and memory between control euploid mice and Ts65Dn mice, but that the learning and memory ability of the Ts65Ds mice could be rescued upon PO administration of PST-001. Furthermore, the data showed no indication of additive or antagonistic effects between PST-001 and the anti-Alzheimer drug memantine, suggesting that the effect of PST-001 acts at different time scale and through different mechanisms than memantine. Obviously, in the absence of in vivo/preclinical evidence of DYRK1A inhibition by PST-001, we cannot guarantee that the pharmacological rescuing of contextual fear conditioning in Ts65Dn mice is necessarily due to DYRK1A inhibition in these mice. Future experiments involving enzyme inhibition or modifications of downstream targets in samples from the treated mouse should provide a key causal link between in vivo/preclinical DYRK1A inhibition and the behavioral effects of PST-001 in this mouse model of DS.

## 4. Materials and Methods

### 4.1. Chemical Preparation

All reagents and solvents were obtained from commercially available sources and were used without further purification. All isolated compounds are >95% pure by HPLC. Procedures for preparing 5-Methoxybenzothiazole as well as original spectra and chromatograms are compiled in the SI-section.

*5-(5-Methoxybenzo[d]thiazol-2-yl)pyridin-3-amine* (**1**). 5-Methoxybenzothiazole (6.34 g, 38.4 mmol), 3-amino-5-bromopyridine (7.41 g, 42.8 mmol), cesium carbonate (12.5 g, 38.4 mmol), copper(I)bromide (1.12 g) and Pd(OAc)_2_ (0.56 g, 2.50 mmol) were suspended in dry DMF (200 mL) under argon. P(*t*-Bu)_3_ (1.00 g, 4.94 mmol) dissolved in 10 mL dry DMF was added. The reaction mixture was heated at 150 °C for 1.5 hrs, cooled to room temperature and poured into EtOAc (100 mL). The organic phase was washed with water (100 mL) and the aqueous phase extracted with EtOAc (2 × 100 mL). The combined organic phase was washed with water, dried (MgSO_4_), filtered and concentrated. Flash chromatography (Heptane: EtOAc 80: 20–50: 50–EtOAc) afforded 4.09 g (41%) of the title compound as a pale yellow solid. Purity >95% (HPLC). ^1^H NMR (300 MHz, DMSO-*d*_6_) δ 8.39 (s, 1H), 8.08 (s, 1H), 8.01 (d, *J* = 8.8, 1H), 7.73–7.49 (m, 2H), 7.11 (dd, *J* = 8.8, 2.5, 1H), 5.71 (s, 2H), 3.87 (s, 3H). MS (pos): 258 (M+H), HR (M+H): 258.0695 (observed), 258.0701 (calculated).
*N-(5-(5-Methoxybenzo[d]thiazol-2-yl)pyridin-3-yl)acetamide* (**PST-001**) [70]. To a suspension of 5-(5-methoxybenzo[d]thiazol-2-yl)pyridin-3-amine (1.29 g, 5.00 mmol) in DCM (25 mL) was added pyridine (10 mL), followed by acetic anhydride (0.95 mL, 10.0 mmol). The reaction mixture was stirred at room temperature overnight, poured into water (100 mL) and the aqueous phase extracted with CHCl_3_: MeOH (90:10) (3 × 100 mL). The combined organic extract was dried (Na_2_SO_4_), filtered and concentrated. The crude material was treated with EtOAc (75 mL), sonicated for 2 min, and filtered. Drying allowed the isolation of 1.30 g (73%) of the title compound as a beige solid from 1.54 g substrate. Purity 99.5% (HPLC). ^1^H NMR (400 MHz, DMSO-*d*_6_) δ 10.41 (s, 1H), 8.88 (s, 1H), 8.80 (s, 2H), 8.03 (d, *J* = 8.8, 1H), 7.66 (d, *J* = 2.3, 1H), 7.13 (dd, *J* = 8.8, 2.4, 1H), 3.87 (s, 3H), 2.13 (s, 3H). ^13^C NMR (150 MHz, DMSO-*d*_6_) δ 169.8, 165.8, 159.5, 155.2, 142.8, 142.2, 136.8, 129.3, 126.7, 123.5, 123.2, 116.3, 106.1, 56.0, 24.4. IR (ATR, cm^−1^): 3301, 1680, 1603, 1551, 1503. MS (pos): 322 (M+Na), HR (M+H): 300.0801 (observed), 300.0807 (calculated).

### 4.2. DYRK1A Protein Production and Crystallization

DYRK1A comprising the kinase domain (DYRK1A, residues 126–490) was produced in bacteria as a HIS-tagged fusion protein and purified as described in detail by Alexeeva et al. [71].

Co-crystallization with the **PST-001** followed the protocol described by Alexeeva et al. In short, the protein kinase DYRK1A was concentrated to 7–10 mg/mL and mixed with the inhibitor solution in DMSO to achieve an approximately 10–50-fold molar excess of the inhibitor. The crystallization solution (100 mM potassium thiocyanate, 50–100 mM NaCl or KCl, 10–16% PEG 3350) gave octahedron-shaped crystals within 5–7 days at room temperature. Crystals were cryoprotected in crystallization solution modified to include 30% ethylene glycol and were flash cooled in liquid nitrogen.

### 4.3. Structure Solution and Refinement

X-ray diffraction data were collected at the Berlin Electron Storage Ring, BESSY II, at the Helmholz Zentrum Berlin, Germany. The images were integrated using the XDSapp 2.0 software [72] and XDS (version 31 January 2020) [73]. The structures were solved by molecular replacement with Phaser [74] using the DYRK1A structure with PDB code 4NCT [71] as search model. The structures were refined by iterative cycles of PHENIX [75] and the CCP4 [76] program REFMAC5 [77] followed by the manual refitting of residues and inhibitors into the electron-density between the refinement cycles and placement of water molecules using Coot v.0.7.2. PRODRG [78] was used to generate the cif-files for inhibitors.

### 4.4. IC_50_ Determination

The principal method utilized is a radioactive filter binding assay using ^33^P ATP [79,80]. The compounds were diluted to the appropriate concentration. The compounds were added to a “mother plate” consisting of samples, controls, and blanks. These serve as the source for “daughter plates” which are stored at −20 °C until assay initiation. Protein Kinases: Enzyme/Substrate mixture was added to the compound, and the compounds were incubated for five minutes at room temperature (RT). ^33^P ATP was added to the compounds to initiate the assay. Orthophosphoric acid was added to the compounds to halt the assay. Assay components were harvested onto P81 filter plates, filter plates were air-dried, scintillation fluid was added to plates, and counts were read on a Topcount NXT. A mean percentage activity was calculated.

### 4.5. Kinase Profile of Benzothiazolylpyridine Derivatives

The determination of protein kinase inhibition was performed at the International Centre for Kinase Profiling at the University of Dundee, UK. The method used is a radioactive filter binding assay using ^33^P ATP as described in the literature [79]. The ATP concentrations were at or below the calculated K_m_ for ATP for each particular kinase.

### 4.6. Luciferin/Luciferase Detected NFAT-Calcineurin Assay of Cellular Effect of DYRK1A

This method was earlier described in Czarna et al. 2018 [81]. For the measurement of the PST-001 inhibitor: HEK293 (ECACC catalog number 85120602) NFAT reporter gen cell lines were cultivated for 24 h without doxycycline before they were stimulated with 1 µM ionomycin and 10 nM PMA for 24 h in presence of the indicated concentration of the PST-001 inhibitor. Renilla luciferase activity was measured in a luminometer. Luciferase activity in the control sample was arbitrary set as 1 and the other values are correlated to this with standard error of the mean (*n* = 3). All experiments were performed in triplicates.

### 4.7. Rat IV Pharmacokinetics

Male Sprague–Dawley rats (surgically prepared with jugular vein cannulation) were administered PST-001 (1 mg/kg; vehicle 10% dimethyl sulfoxide 90% 2-hydroxypropyl-β-cyclodextrin (20% *w*/*v* in PBS)) intravenously by bolus to tail vein at a dose volume of 1 mL/kg. For determining plasma pharmacokinetics blood samples (via cardiac puncture into heparinized tubes and centrifuged for plasma at 4 °C) were serially sampled via jugular vein at 2, 0.5, 5, 15, and 30 min, 1, 2, 4, 8, and 24 h timepoints post dosing. Three animals per timepoint were sampled. All samples were stored at −20 °C pending analysis. Bioanalysis was carried out by UHPLC-TOF mass spectrometry using electrospray ionization against matrix matched calibration lines. The pharmacokinetic parameters were subsequently calculated by noncompartmental methods.

### 4.8. Mouse PO Pharmacokinetics

Male C57BL/6 mice were administered PST-001 (5 mg/kg; vehicle 10% dimethyl sulfoxide 90% 2-hydroxypropyl-β-cyclodextrin (20% *w*/*v* in PBS)) orally by gavage at a dose volume of 10 mL/kg. For determining plasma and brain pharmacokinetics blood samples (via cardiac puncture into heparinized tubes and centrifuged for plasma at 4 °C) and brains were terminally sampled at 0.25, 0.5, 1, 2, 3, 4, 6, and 8 h timepoints post dosing. Three animals per timepoint were sampled. The three animals designated for the 8 h timepoint were housed in a metabolism cage post-dosing for collection of urine. All samples were stored at −20 °C pending analysis. Bioanalysis was carried out by UHPLC-TOF mass spectrometry using electrospray ionization against matrix matched calibration lines.

The pharmacokinetic parameters were subsequently calculated by noncompartmental methods.

### 4.9. Bioanalysis

Samples are prepared by protein precipitation and/or dilution: tissues are homogenized first. Analysis is carried out using a generic internal standard, with matrix-matched calibration standards bracketing the study samples.

Samples were analyzed by high resolution accurate mass UHPLC-TOF MS. The LC-MS system comprised an Agilent 1290 Infinity UHPLC pump with an Agilent 1290 Infinity HTS autosampler, coupled with an Agilent 6550 iFunnel QToF mass spectrometer, equipped with a Waters Acquity BEH Phenyl UPLC column, (50 × 2.1) mm, 1.7 µm particle size. The mass spectrometer was operated in full scan mode, with positive ion electrospray data acquired over the *m*/*z* mass range 100–1000.

### 4.10. Ts65Ds Model of Cognition. Determination of Blood, Liver, and Brain PST-001 Concentrations

To determine the necessary amount of added PST-001 in food to reach a serum concentration consistent with the DYRK1A inhibitory potency of this compound, a GC/MS-based procedure was used. Six control euploid mice of the same genetic background as the Ts65Dn mice were used to perform this analysis; two mice per dose and three doses tested [82]. An adapted LC/MS protocol was used for concentration determination of PST-001 in various murine tissues, such as liver and brain.

### 4.11. Ts65Ds Model of Cognition. Contextual Fear Conditioning

The capacity for contextual memory of 10- to 16-week-old Ts65Dn mice was compared with age-matched euploid control mice using the same fear conditioning protocol used in a previous work with the drug memantine [40]. Briefly, the mice were exposed for 3 min to the context (Med Associates, St. Albans, VT, Modular Mouse Test Chamber) serving as a conditioning stimulus, followed by an electric shock (2 s, 0.7 mA, constant electric current) representing the unconditioned stimulus. The memory test was performed 24 h later, by re-exposing the mice to the same context. Freezing, defined as a species-specific defensive reaction characterized by lack of movement besides respiration and heartbeat, associated with crouching posture, was used as a measure of learning, and assessed for 180 s, every 9 s by two trained observers aware of the mouse genotypes and the pharmacological agent being used. The mean number of observations indicating freezing behavior was expressed as percentage of freezing in relation to the total number of observations. All experiments were video-recorded, for automated video-analysis, confirmation of the trained-observer scores, and archival purposes.

Prior to contextual fear conditioning experiments, all mice were exposed for three-days to either food mixture alone or food mixture with added PST-001. In addition, the mice continued to consume these same food preparations during the two days necessary to perform the behavioral testing. For the chronic administration of PST-001, the following groups were used: (1) euploid control mice exposed to food mixture with no PST-001 (*n* = 11); (2) control mice exposed to food mixture with PST-001 (*n* = 10); (3) Ts65Dn mice exposed to food mixture with no PST-001 (*n* = 10); and (4) Ts65Dn mice exposed to food mixture with PST-001 (*n* = 11). These animals were killed after the experiments, and their livers and brains were harvested for PST-001 concentration determinations. Comparisons between group means were done with Two-way factorial ANOVA, and Fisher protected least significant difference (PLSD) post hoc test was performed for all significant ANOVA (using Statistica version 13, Tibco Software, Palo Alto, CA, USA).

## 5. Conclusions

PST-001 is an effective and selective DYRK1A inhibitor with proven peroral bioavailability in rodent models. The PK-profile of PST-001 is characterized and sufficiently good to enable clear effects in an animal model. The compound should hence be a valuable addition to the arsenal of DYRK1A inhibitors compatible with in vivo studies, in particular where selectivity towards GSK3β is required.

## Figures and Tables

**Table 1:** Percent remaining inhibitory activity at high and low PST-001 concentration on a panel of protein kinases. The full profile data set is compiled in the Supplementary Information (Table S2).

Kinase	% Remaining Activity	
	100 μM	1 μM
DYRK1A	3	8
DYRK2	4	21
DYRK3	5	20
Aurora A	136	133
Aurora B	24	106
GSK3β	100	107
ERK8	28	89
CLK2	5	22

**Table 2:** In vitro pharmacokinetic (PK) assessment of PST-001 in rat (male Sprague Dowly) or mouse (male C57Bl) following IV or PO administration.

PK Parameter			Administration Route and Measured Tissue		
	IV (Plasma) (Rat)	IV Plasma (Mouse)	IV Brain (Mouse)	PO Plasma (Mouse)	PO Brain (Mouse)
PST-001 dose	1 mg/kg	1 mg/kg	1 mg/kg	5 mg/kg	5 mg/kg
t_½_ (h)	0.72	0.15	0.15	NQ	0.27
T_max_ (h)	0.03	0.08	0.08	1.00	0.25
C_max_ (ng/mL)	647	362	867	76	153
AUC_all_ (h ∗ ng/mL)	158	84	200	89	189
CL (mL/h/kg)	6281	11865	4989		
Vd (mL/kg)	2387	1388	553		
f				21.2%	

## Data Availability

The atomic co-ordinates and structure factors for DYRK1A with the inhibitor PST-001 has been deposited in the Protein Data Bank under PDB accession code 6YF8 https://www.rcsb.org/structure/6YF8.

## Supplementary Materials

The following are available online at https://www.mdpi.com/article/10.3390/ph14111170/s1, Figure S1: ^1^H NMR spectrum (DMSO-d_6_) of 1-(3-Methoxyphenyl)thiourea, Figure S2: ^1^H NMR spectrum (DMSO-d_6_) of 5-Methoxybenzo[d]thiazol-2-amine, Figure S3: ^1^H NMR spectrum (CDCl_3_) of 5-Methoxybenzo[d]thiazole, Figure S4: ^1^H NMR spectrum (DMSO-d_6_) of 5-(5-methoxybenzo[d]thiazol-2-yl)pyridin-3-amine (**1**), Figure S5: ^1^H NMR spectrum (DMSO-d_6_) of *N*-(5-(5-Methoxybenzo[d]thiazol-2-yl)pyridin-3-yl)acetamide (**PST-001**), Figure S6: HPLC-chromatogram of *N*-(5-(5-Methoxybenzo[d]thiazol-2-yl)pyridin-3-yl)acetamide (**PST-001**) showing a purity of >99%. Table S1: Crystallographic data and model statistics for 6YF8., Table S2: Remaining protein kinase activity after exposure to PST-001 and compound 1. Two concentrations of the inhibitor were used, 100 and 1 μM.
