# Supplementary material for: Novel DYRK1A Inhibitor Rescues Learning and Memory Deficits in a Mouse Model of Down Syndrome
Source: Pharmaceuticals (Basel). 2021 Nov 17;14(11):1170. doi: 10.3390/ph14111170 (PMC8617627; doi:10.3390/ph14111170)
Supplement: Supplementary file 1 [file pharmaceuticals-14-01170-s001.zip › pharmaceuticals-1427388-supplementary.pdf]

Novel DYRK1A Inhibitor Rescues Learning and Memory Deficits in a Mouse Model of Down Syndrome

Wenche Stensen [1,2], Ulli Rothweiler [1,2], Richard Alan Engh [1], Melissa R. Stasko [3], Ilya Bederman [3], Alberto C. S. Costa [3], Anders Fugelli [2] and John S. Mjøen Svendsen [1,2,*]

[1] Department of Chemistry, UiT, The Arctic University of Norway, 9037 Tromsø, Norway; wenche.stensen@uit.no (W.S.); ulli.rothweiler@uit.no (U.R.); richard.engh@uit.no (R.A.E.)
[2] Pharmasum Therapeutics AS, Gaustadalleen 21, 0349 Oslo, Norway; afugelli@pharmasum.com
[3] Departments of Pediatrics, Psychiatry, Macromolecular Science and Engineering, Case Western Reserve University, 11100 Euclid Avenue, Cleveland, OH 44106-6090, USA; mxs1081@case.edu (M.R.S.); irb2@case.edu (I.B.); alberto.costa@case.edu (A.C.S.C.)
* Correspondence: john-sigurd.svendsen@uit.no

Preparation of PST-001

1-(3-Methoxyphenyl)thiourea

A mixture of sodium thiocyanate (49.38 g, 609 mmol) in isopropyl acetate (400 ml) was added m-anisidine (45.6 ml, 406 mmol) at room temperature. TFA (78 ml, 1019 mmol) was then added dropwise slowly ensuring that the temperature of the reaction mixture was maintained at 40 °C or less. After complete addition the reaction mixture was stirred at 85 °C for 16.5 hrs. The mixture was cooled to room temperature and HPLC-water (50 ml) was added before further cooling to 0 °C. The crude product was isolated by vacuum filtration on a sintered glass funnel and washed with HPLC-water (50 ml) and dried under reduced pressure to afford 60.5 g (82 %) of the crude title compound as a colorless powder which was used in the next step without further purification.

^{1}H NMR (300 MHz, DMSO-d_6) δ 9.68 (s, 1H), 7.48 (bs, 2H), 7.22 (t, *J* = 8.1, 1H), 7.12 (s, 1H), 6.92 (d, *J* = 8.0, 1H), 6.69 (dd, *J* = 8.3, 1.7, 1H), 3.73 (s, 3H).

MS (pos): 205 (M+Na), 183 (M+H)

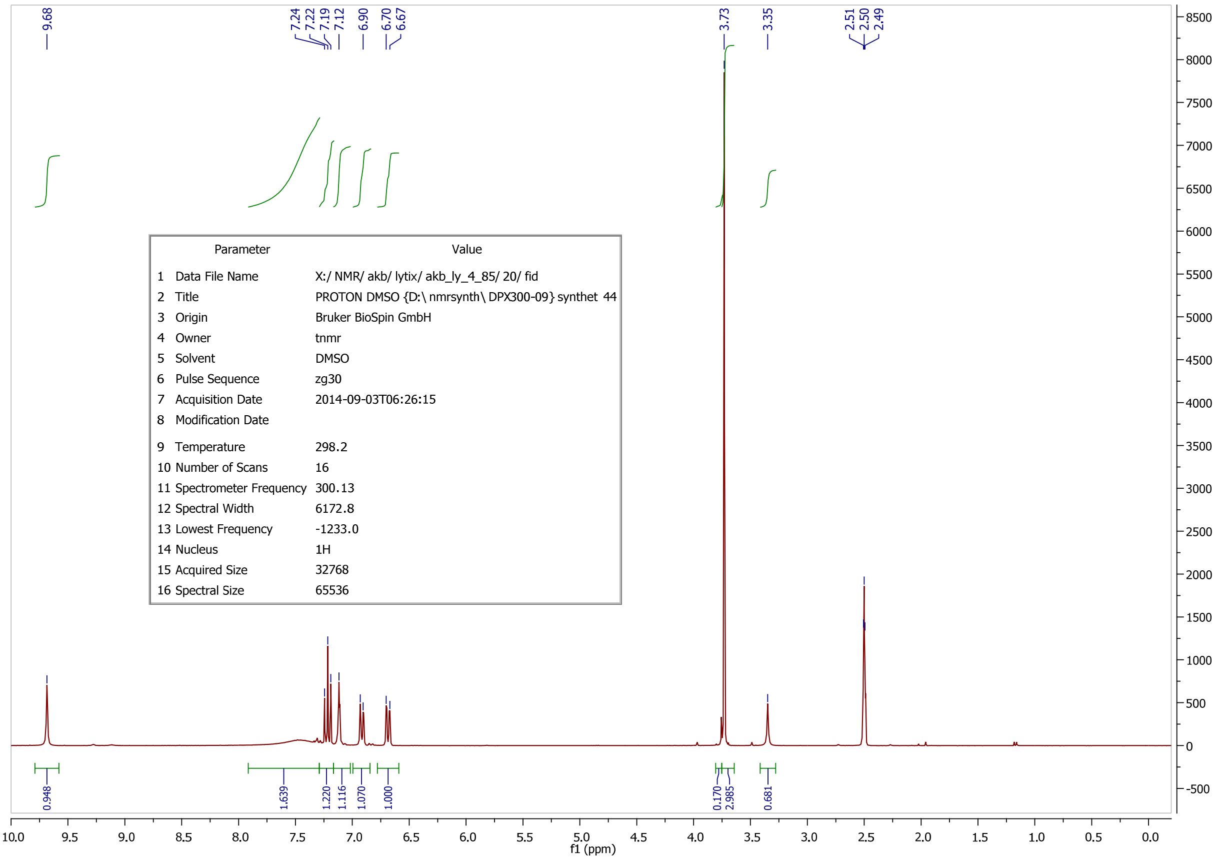

Figure S1. ^{1}H NMR spectrum (DMSO-d_6) of 1-(3-Methoxyphenyl)thiourea.

5-Methoxybenzo[*d*]thiazol-2-amine

A suspension of 1-(3-methoxyphenyl)thiourea (60.39 g, 331.4 mmol) and LiBr (43.24 g, 497.9 mmol) in acetic acid (600 ml) was added Br_2 (17 ml, 330.8 mmol) slowly drop wise / portion wise ensuring that the temperature of the reaction mixture was maintained at 33 °C or less. After complete addition the reaction mixture was stirred at 40 °C for 18.5 hrs. The reaction mixture was cooled to 0 °C before the crude product was isolated by vacuum filtration on a sintered glass funnel and washed with 5 % Na_2CO_3 (aq) (4 x 100 ml) and HPLC-water (100 ml x 2) and dried under reduced pressure to afford 31.72 g of the crude title compound as a colorless powder. The washings were added 2 M NaOH (aq) to pH ~ 11 and extracted with CH_2Cl_2 (3 x 500 ml), dried (Na_2SO_4), filtered and concentrated to afford additional 7.22 g of the impure title compound. The concentrated extracts were purified by flash-chromatography on silica gel (400 g) eluting with CH_2Cl_2 – CH_2Cl_2 (100:2) to afford 4.02 g of the title compound. Total yield 35.74 g (60 %).

^1H NMR (400 MHz, DMSO-d_6) δ 7.56 (bs, 2H), 7.50 (d, J = 8.6, 1H), 6.92 (d, J = 2.5, 1H), 6.65 (dd, J = 8.6, 2.5, 1H), 3.75 (s, 3H).

MS (pos): 181 (M+H)

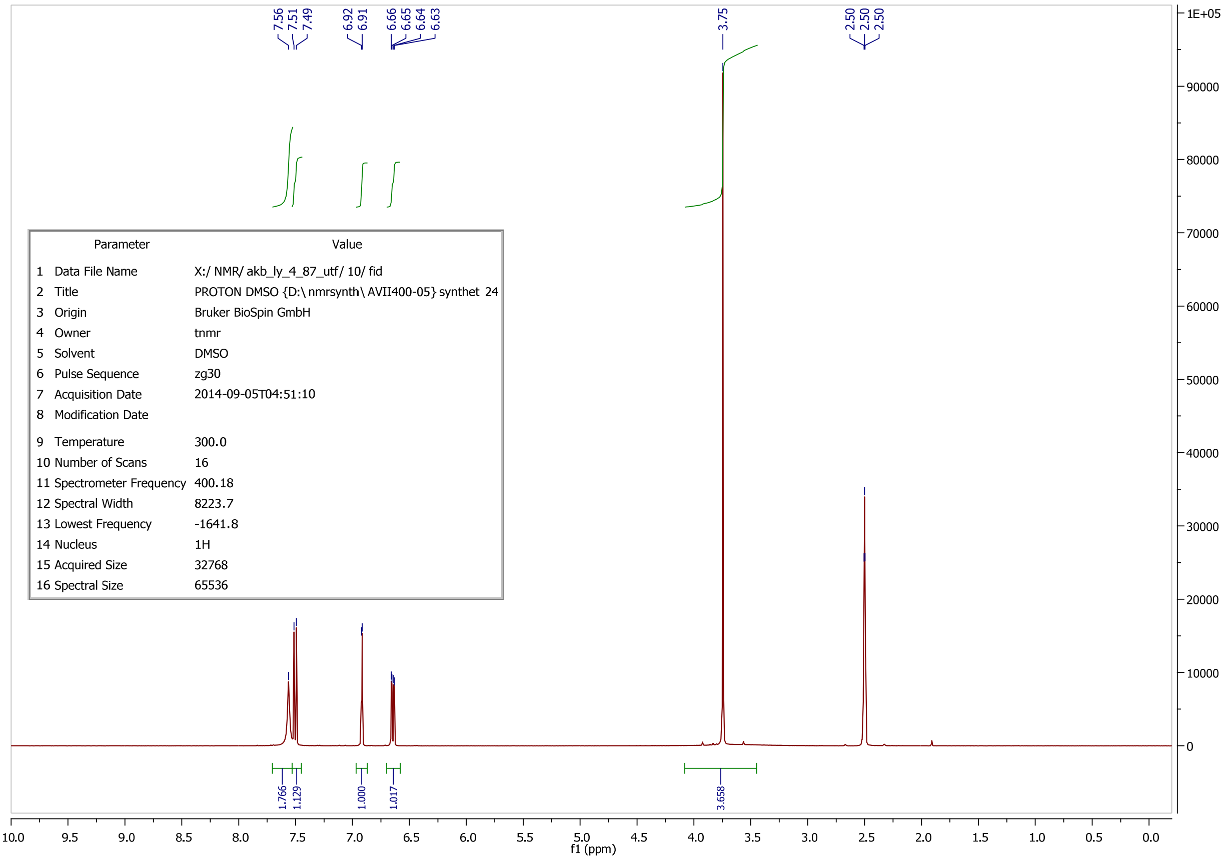

Figure S2. ^1H NMR spectrum (DMSO-d_6) of 5-Methoxybenzo[d]thiazol-2-amine.

5-Methoxybenzo[*d*]thiazole

A mixture of 5-methoxybenzo[*d*]thiazol-2-amine (35.69 g, 198 mmol) in dry DMF (290 ml) was added drop wise over 55 minutes to a solution of *t*-butyl nitrite (42 ml, 353 mmol) at 65 °C. The reaction mixture was stirred at 65 °C for 40 minutes, cooled to room temperature and poured into 1 M HCl (1 L). The mixture was extracted with EtOAc (4 x 500 ml), dried (Na_2SO_4), filtered and concentrated under reduced pressure. Dry-flash chromatography on silica gel (1 kg) eluting with heptane – heptane:EtOAc (80:20) afforded 14.09 g (43 %) of the title compound as a yellow oil with some solidified material.

^{1}H NMR (300 MHz, $CDCl_3$) δ 8.95 (s, 1H), 7.78 (d, *J* = 8.8, 1H), 7.59 (d, *J* = 2.5, 1H), 7.08 (dd, *J* = 8.7, 2.3, 1H), 3.88 (s, 3H).

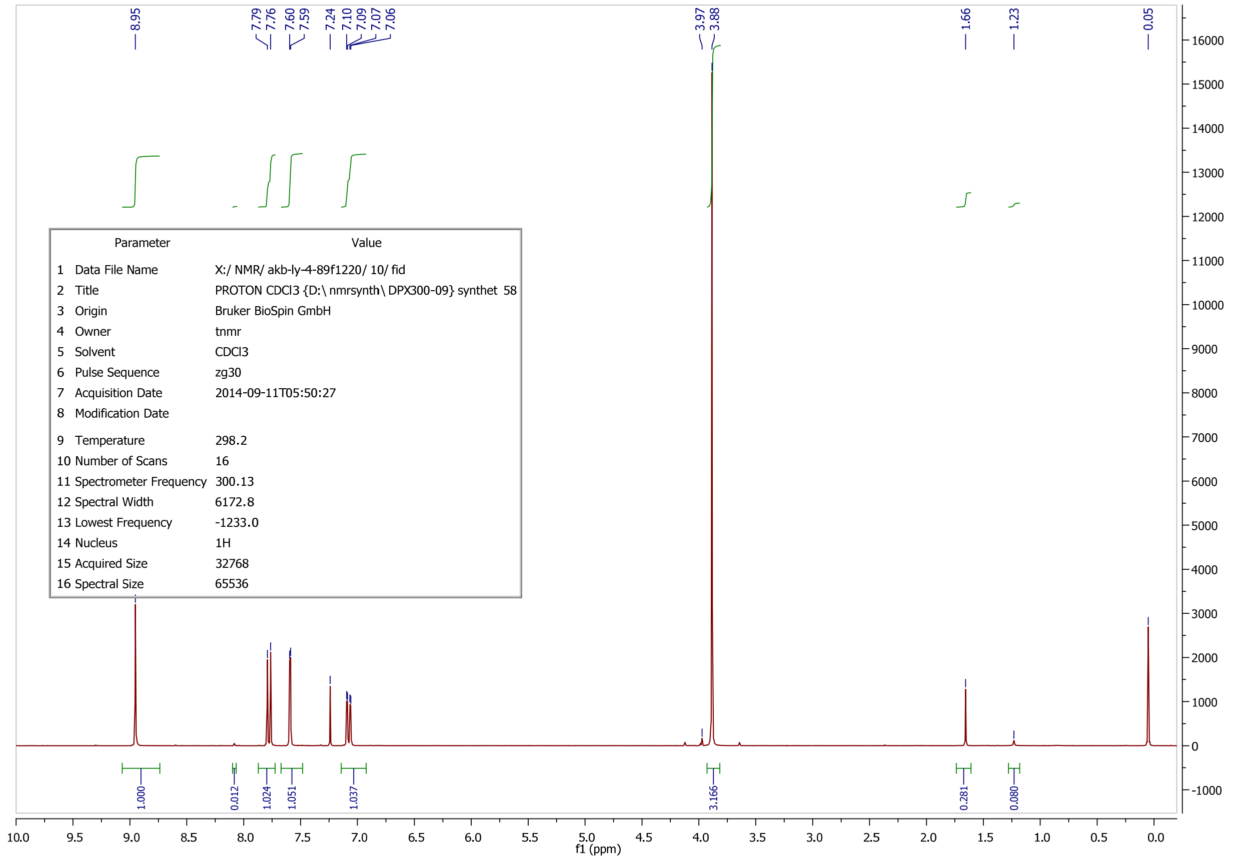

Figure S3. ^{1}H NMR spectrum ($CDCl_3$) of 5-Methoxybenzo[d]thiazole.

5-(5-methoxybenzo[d]thiazol-2-yl)pyridin-3-amine (1)

5-Methoxybenzothiazole (6.34 g, 38.4 mmol), 3-amino-5-bromopyridine (7.41 g, 42.8 mmol), cesium carbonate (12.5 g, 38.4 mmol), copper(I)bromide (1.12 g) and $Pd(OAc)_2$ (0.56 g, 2.50 mmol) were suspended in dry DMF (200 ml) under argon. P(*t*-Bu)$_3$ (1.00 g, 4.94 mmol) dissolved in 10 ml dry DMF was added. The reaction mixture was heated at 150 °C for 1.5 hrs, cooled to room temperature and poured into EtOAc (100 ml). The organic phase was washed with water (100 ml) and the aqueous phase extracted with EtOAc (2 x 100 ml). The combined organic phase was washed with water, dried ($MgSO_4$), filtered and concentrated. Flash chromatography (Heptane : EtOAc 80 : 20 – 50 : 50 – EtOAc) afforded 4.09 g (41%) of the title compound as a pale yellow solid.

^{1}H NMR (300 MHz, DMSO-*d*$_6$) δ 8.39 (s, 1H), 8.08 (s, 1H), 8.01 (d, *J* = 8.8, 1H), 7.73 – 7.49 (m, 2H), 7.11 (dd, *J* = 8.8, 2.5, 1H), 5.71 (s, 2H), 3.87 (s, 3H).

MS (pos): 258 (M+H)

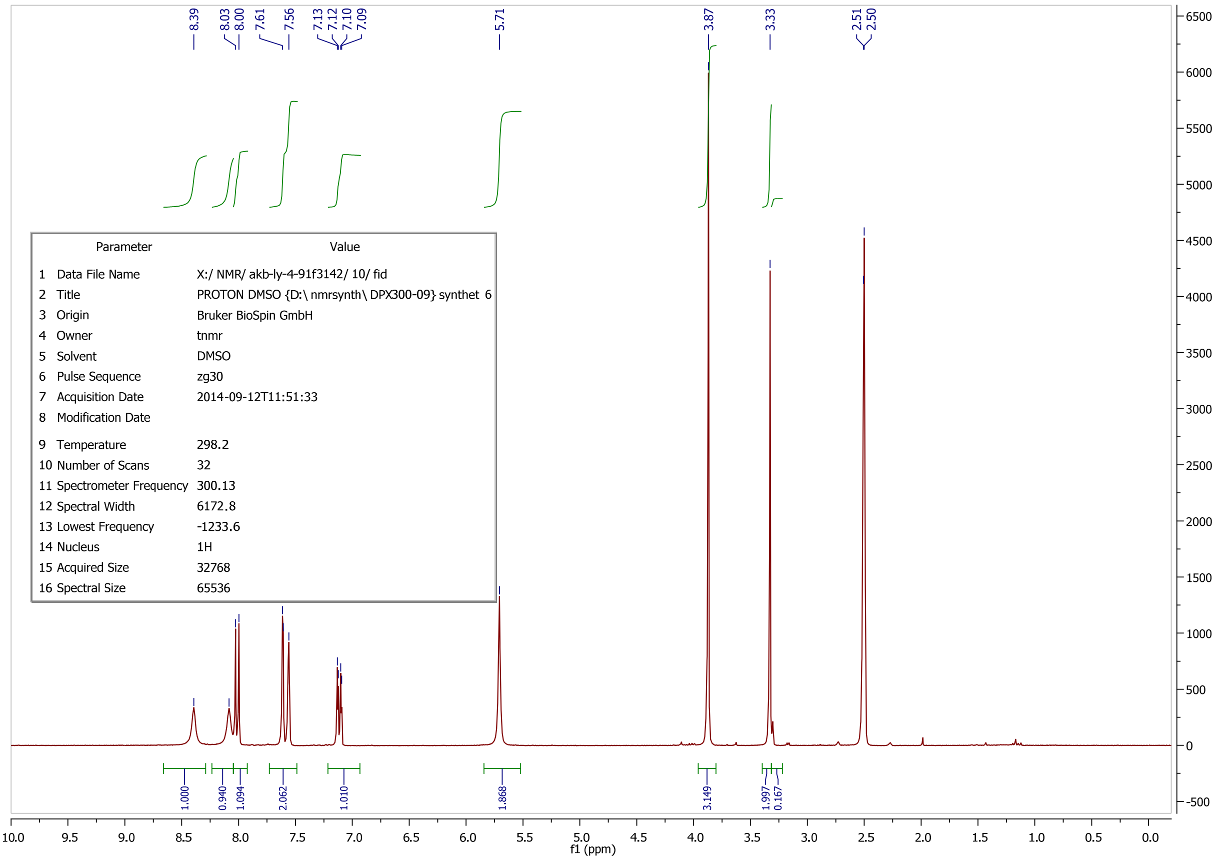

Figure S4. ^{1}H NMR spectrum (DMSO-d$_6$) of 5-(5-methoxybenzo[d]thiazol-2-yl)pyridin-3-amine (**1**).

N-(5-(5-Methoxybenzo[d]thiazol-2-yl)pyridin-3-yl)acetamide (PST-001)

To a suspension of 5-(5-methoxybenzo[d]thiazol-2-yl)pyridin-3-amine (1.29 g, 5.00 mmol) in DCM (25 ml) was added pyridine (10 ml), followed by acetic anhydride (0.95 ml, 10.0 mmol). The reaction mixture was stirred at room temperature overnight, poured into water (100 ml) and the aqueous phase extracted with $CHCl_3$: MeOH (90:10) (3 x 100 ml). The combined organic extract was dried (Na_2SO_4), filtered and concentrated. The crude material was treated with EtOAc (75 ml), sonicated for 2 minutes and filtered. Drying allowed the isolation of 1.30 gram (73%) of the title compound as a beige solid from 1.54 g substrate.

^{1}H NMR (400 MHz, DMSO-d_6) δ 10.41 (s, 1H), 8.88 (s, 1H), 8.80 (s, 2H), 8.03 (d, J = 8.8, 1H), 7.66 (d, J = 2.3, 1H), 7.13 (dd, J = 8.8, 2.4, 1H), 3.87 (s, 3H), 2.13 (s, 3H).

MS (pos): 322 (M+Na).

HPLC (234 nm): 99.5 % (area-%).

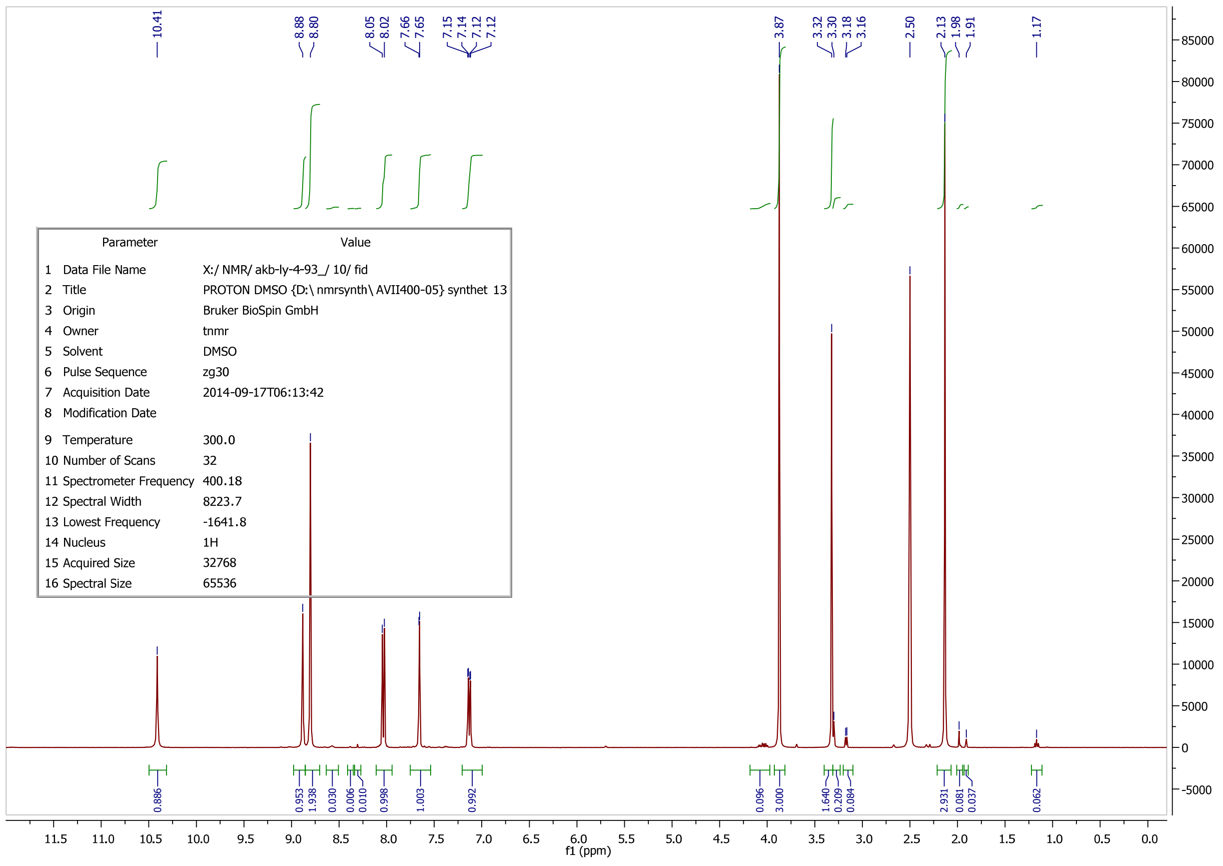

Figure S5. ^{1}H NMR spectrum (DMSO-d_6) of N-(5-(5-Methoxybenzo[d]thiazol-2-yl)pyridin-3-yl)acetamide (**PST-001**).

Sample Name: AKB_LY_4_93_LCV

1

Sample Name : AKB_LY_4_93_LCV
Injection Date : Tue, 16. Sep. 2014
Acq. Method : AKB_LYTIX.M
Inj. Vol. : 1 µl
Acq Operator : AKB
Data file : D:\CHEM32\1\DATA\AKB_LY\AKB_LY_4_93_LCV.D

synthetica

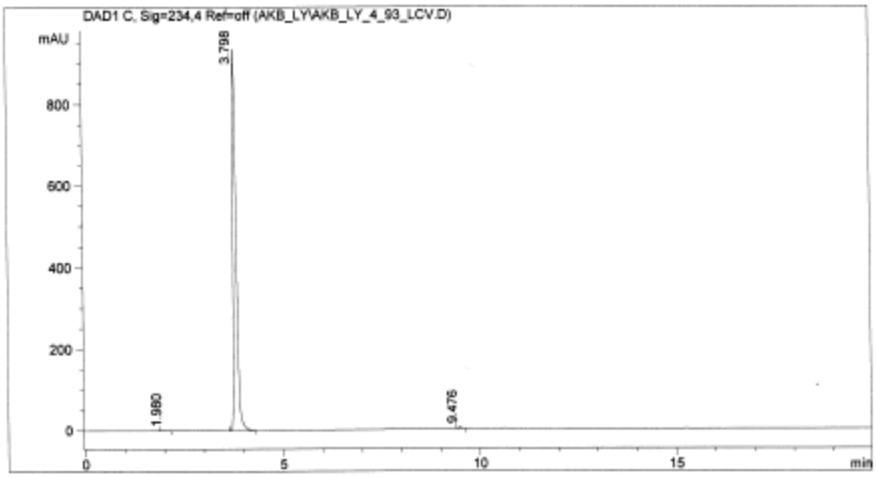

Signal 1: DAD1 C, Sig=234,4 Ref=off

Peak #	RT [min]	Type	Width [min]	Area	Area %	Height	Name
1	1.980	BB	0.105	7.419	0.139	1.066	
2	3.798	BB	0.086	5291.513	99.471	936.866	
3	9.476	BB	0.092	20.733	0.390	3.538	

*** End of Report ***

Figure S6. HPLC-chromatogram of *N*-(5-(5-Methoxybenzo[d]thiazol-2-yl)pyridin-3-yl)acetamide (**PST-001**) showing a purity of >99%.

DYRK1A protein production and crystallization

Table S1. Crystallographic data and model statistics for 6YF8

Compound	**PST001**
PDB	6YF8
**DATA COLLECTION**	
Synchrotron radiation	BESSY II 14.2
Detector	Pilatus
Wavelength, Å	0.918400
Number of frames	1800
Oscillation range/frame	0.1
**DIFFRACTION DATA**	
Space group	$P2_12_12_1$
Unit cell parameters, Å	88.0 88.04 229.72
Protein molecules in AU	4

Number of reflections	200855
Unique reflections	30124
Resolution range , Å (final shell)	50.0 - 3.2 (3.39 - 3.2)
Completeness (final shell) %	99.3 (98.4)
I/ (final shell)	7.02 (1.36)
R factor observed %	26.8 (130.9)
CC1/2 (final shell)	99.1 (58.6)
**REFINEMENT**	
Resolution limits, Å	48.3 - 3.2
Number of used reflections	30122
Data completeness	99.37
Percentage of free reflections	4.8
Number of protein atoms	10930
Number of heterogen atoms	84
Number of water	0
R factor overall/free	0.25 / 0.28
Wilson B-factor (Å2)	66.8
RMS bonds/angles	0.003/0.75
Ramachandran (favored / allowed / outliers %)	95 / 4.4 / 0.6

Table S2. Remaining protein kinase activity after exposure to **PST-001** and compound **1**. Two concentrations of the inhibitor were used, 100 and 1 μM.

Compounds	**PST-001**		**1**	
Tested concentration (μM)	100	1	100	1
MKK1	135	117	84	101
MKK2	100	75	62	81
MKK6	115	140	118	120
ERK1	108	109	81	99
ERK2	112	115	96	102
ERK5	51	72	37	66
JNK1	130	113	94	112
JNK2	129	126	104	123
JNK3	122	127	61	125
p38a MAPK	106	110	96	103
p38b MAPK	115	107	101	119
p38g MAPK	105	125	37	110

Compounds	**PST-001**		**1**	
Tested concentration (μM)	100	1	100	1
p38d MAPK	112	115	95	116
ERK8	28	89	4	86
RSK1	88	99	42	99
RSK2	100	113	56	115
PDK1	105	110	67	104
PKBa	106	116	72	120
PKBb	98	112	15	123
SGK1	82	102	62	99
S6K1	81	113	58	118
PKA	88	88	74	98
ROCK 2	64	110	5	108
PRK2	101	102	74	103

Compounds	PST-001		1	
Tested concentration (μM)	100	1	100	1
PKCa	85	119	63	110
PKCγ	124	125	61	115
PKCz	124	119	128	106
PKD1	92	103	23	94
STK33	112	113	57	111
MSK1	64	104	60	109
MNK1	94	111	20	97
MNK2	66	106	27	90
MAPKAP-K2	111	109	66	122
MAPKAP-K3	113	115	74	115
PRAK	116	114	36	128
CAMKKb	54	104	37	107
CAMK1	100	107	52	99
SmMLCK	99	118	41	98
PHK	83	107	52	101
DAPK1	101	114	58	103
CHK1	115	104	86	101
CHK2	85	105	52	114
GSK3b	100	107	25	107
CDK2-Cyclin A	69	110	19	96
CDK9-Cyclin T1	120	122	83	113
PLK1	115	112	73	99
Aurora A	136	133	97	132
Aurora B	24	106	21	107
TLK1	134	129	115	131
LKB1	150	123	161	109
AMPK (hum)	69	99	46	107
MARK1	110	122	69	122
MARK2	106	116	62	112
MARK3	95	99	34	98
MARK4	103	121	58	131
BRSK1	111	114	37	115
BRSK2	125	128	53	125
MELK	44	99	12	96
NUAK1	107	130	35	112

Compounds	PST-001		1	
Tested concentration (μM)	100	1	100	1
SIK2	65	112	10	102
SIK3	78	114	19	106
TSSK1	114	124	70	107
CK1γ2	101	114	24	108
CK1δ	59	99	13	107
CK2	95	102	21	103
TTBK1	110	109	90	109
TTBK2	117	119	103	123
DYRK1A	3	8	0	7
DYRK2	4	21	1	11
DYRK3	5	20	2	14
NEK2a	107	119	51	111
NEK6	113	112	115	106
IKKb	108	111	38	105
IKKe	122	105	55	110
TBK1	104	119	60	105
PIM1	32	98	24	87
PIM2	102	120	36	110
PIM3	76	93	24	90
SRPK1	108	115	55	108
EF2K	106	113	84	112
EIF2AK3	118	123	84	122
HIPK1	113	113	36	116
HIPK2	77	90	10	79
HIPK3	100	123	28	120
CLK2	5	22	1	23
PAK2	122	114	103	110
PAK4	98	116	54	109
PAK5	129	113	70	111
PAK6	113	112	80	108
MST2	54	120	32	124
MST3	113	118	96	114
MST4	113	112	72	101
GCK	123	118	28	105
MAP4K3	94	111	22	98

Compounds	PST-001		1	
Tested concentration (μM)	100	1	100	1
MAP4K5	92	82	61	106
MINK1	88	104	36	108
MEKK1	109	126	107	132
MLK1	96	108	16	106
MLK3	60	109	29	101
TESK1	85	105	28	110
TAO1	111	102	57	97
ASK1	131	114	150	116
TAK1	63	48	6	70
IRAK1	93	103	24	95
IRAK4	32	88	18	85
RIPK2	20	109	20	104
OSR1	136	120	111	128
TTK	69	88	47	86
MPSK1	113	121	106	110
WNK1	118	127	113	116
ULK1	120	129	77	116
ULK2	119	111	50	101
TGFBR1	129	112	118	105
Src	106	100	42	123
Lck	100	93	52	106
CSK	126	121	78	116
YES1	128	130	58	122

Compounds	PST-001		1	
Tested concentration (μM)	100	1	100	1
ABL	99	97	46	108
BTK	86	92	31	101
JAK2	125	123	55	117
SYK	58	112	38	113
ZAP70	131	123	63	119
TIE2	133	129	85	135
BRK	106	106	50	94
EPH-A2	119	124	90	112
EPH-A4	106	117	85	89
EPH-B1	108	117	91	122
EPH-B2	125	137	96	136
EPH-B3	130	147	71	130
EPH-B4	130	107	97	112
FGF-R1	107	107	91	110
HER4	94	103	16	104
IGF-1R	104	104	68	96
IR	120	116	45	108
IRR	120	117	77	113
TrkA	45	111	33	104
DDR2	80	98	49	107
VEG-FR	41	111	20	110
PDGFRA	73	100	26	101
PINK	118	119	114	121
